# Supplementary material for: Precipitation legacy effects on soil microbiota facilitate adaptive drought responses in plants
Source: Nat Microbiol. 2025 Oct 30;10(11):2823–44. doi: 10.1038/s41564-025-02148-8 (PMC12578634; doi:10.1038/s41564-025-02148-8)
Supplement: Supplementary file 2 — Reporting Summary [file 41564_2025_2148_MOESM2_ESM.pdf]

nature portfolio

Corresponding author(s): Gabriel Castrillo
Maggie R. Wagner

Last updated by author(s): 27 August 2025

Reporting Summary

Nature Portfolio wishes to improve the reproducibility of the work that we publish. This form provides structure for consistency and transparency in reporting. For further information on Nature Portfolio policies, see our Editorial Policies and the Editorial Policy Checklist.

Statistics

For all statistical analyses, confirm that the following items are present in the figure legend, table legend, main text, or Methods section.

n/a	Confirmed	
☐	☒	The exact sample size (n) for each experimental group/condition, given as a discrete number and unit of measurement
☐	☒	A statement on whether measurements were taken from distinct samples or whether the same sample was measured repeatedly
☐	☒	The statistical test(s) used AND whether they are one- or two-sided<br>*Only common tests should be described solely by name; describe more complex techniques in the Methods section.*
☐	☒	A description of all covariates tested
☐	☒	A description of any assumptions or corrections, such as tests of normality and adjustment for multiple comparisons
☐	☒	A full description of the statistical parameters including central tendency (e.g. means) or other basic estimates (e.g. regression coefficient) AND variation (e.g. standard deviation) or associated estimates of uncertainty (e.g. confidence intervals)
☐	☒	For null hypothesis testing, the test statistic (e.g. F, t, r) with confidence intervals, effect sizes, degrees of freedom and P value noted<br>*Give P values as exact values whenever suitable.*
☒	☐	For Bayesian analysis, information on the choice of priors and Markov chain Monte Carlo settings
☐	☒	For hierarchical and complex designs, identification of the appropriate level for tests and full reporting of outcomes
☐	☒	Estimates of effect sizes (e.g. Cohen's d, Pearson's r), indicating how they were calculated

Our web collection on statistics for biologists contains articles on many of the points above.

Software and code

Policy information about availability of computer code

Data collection	Software packages are detailed in the Methods section.
Data analysis	Software packages are detailed in the Methods section. We deposited all scripts and source data required to reproduce the results of this study in the following Zenodo repository: doi: 10.5281/zenodo.13821005.

For manuscripts utilizing custom algorithms or software that are central to the research but not yet described in published literature, software must be made available to editors and reviewers. We strongly encourage code deposition in a community repository (e.g. GitHub). See the Nature Portfolio guidelines for submitting code & software for further information.

Data

Policy information about availability of data

All manuscripts must include a data availability statement. This statement should provide the following information, where applicable:

- Accession codes, unique identifiers, or web links for publicly available datasets
- A description of any restrictions on data availability
- For clinical datasets or third party data, please ensure that the statement adheres to our policy

The 16S rRNA gene amplicon sequenng data, shotgun metagenomic data, and metatranscriptome data associated with this study have been deposited in the NCBI Sequence Read Archive under the BioProject IDs PRJNA1267293, PRJNA1267715, PRJNA1268489, and PRJNA1186942. The raw RNA-seq data from gamagrass and

maize have been deposited in the Gene Expression Omnibus under accessions GSE282586 and GSE282587, respectively. Plant phenotype data and soil data are available in a Zenodo repository.

Research involving human participants, their data, or biological material

Policy information about studies with human participants or human data. See also policy information about sex, gender (identity/presentation), and sexual orientation and race, ethnicity and racism.

Reporting on sex and gender	N/A
Reporting on race, ethnicity, or other socially relevant groupings	N/A
Population characteristics	N/A
Recruitment	N/A
Ethics oversight	N/A

Note that full information on the approval of the study protocol must also be provided in the manuscript.

Field-specific reporting

Please select the one below that is the best fit for your research. If you are not sure, read the appropriate sections before making your selection.

☐ Life sciences ☐ Behavioural & social sciences ☒ Ecological, evolutionary & environmental sciences

For a reference copy of the document with all sections, see nature.com/documents/nr-reporting-summary-flat.pdf

Ecological, evolutionary & environmental sciences study design

All studies must disclose on these points even when the disclosure is negative.

Study description	Six soils from Kansas, USA were collected and thoroughly characterized using shotgun metagenome and metatranscriptome sequencing, as well as X-ray CT scanning to quantify porosity. The soils were then exposed to drought treatment or well-watered conditions, with or without a plant host (Tripsacum dactyloides) in a factorial design. In the "Conditioning Phase", a total of 192 sterile 100 mL pots were filled with the six soils and which were then randomly assigned to one of four conditions in a fully-factorial design: with or without a host, and either water-stressed or well-watered. Half the pots were planted with seedlings of the native prairie grass T. dactyloides (Eastern gamagrass, cultivar "Pete"); the rest remained unplanted. Thus, the replication was N=8 per soil per treatment. In the "Test Phase", the microbial extract from each of the 192 Conditioning Phase pots (as well as the 24 uninoculated control pots) was used to inoculate 4 pots for the "Test Phase": one pot per combination of watering treatment (droughted or control) and host species (maize or gamagrass).
Research sample	Six soils were collected from never-plowed prairie remnants in Kansas, USA. All of the plants measured in this study were grown from seed in growth chambers.
Sampling strategy	The collection sites were selected to be evenly spaced across the precipitation gradient. Six independent sub-samples were randomly selected within each of the six sites.
Data collection	Numerous methods of data collection were used and are detailed in the Methods section of the Supplementary Information. Data collection and recording was done by Nichole Ginnan, Valeria Custodio, and several core facilities.
Timing and spatial scale	The soils were collected during a single collection trip, i.e., they represent a "snapshot" in time (October 2020). The spatial scale is on the order of kilometers and is detailed in the manuscript.
Data exclusions	No data were excluded from the analysis other than a couple of outlier data points that were clear errors, e.g., biologically impossible data points such as a root that was recorded as 100% aerenchyma.
Reproducibility	For the "Test Phase" analyses, we grouped together low-precipitation and high-precipitation soils so that our experiment was effectively replicated over three distinct soils representing each precipitation category.
Randomization	We used a random number generator within an Excel spreadsheet to randomize the placement of plants within the growth chamber with respect to microbial inoculum and drought treatment. We did the same for the soil mesocosms (randomized with respect to starting soil, drought treatment, and presence or absence of a host plant).
Blinding	Soil mesocosms and plants were tracked using a non-descriptive ID number. During the experiments, we were not blinded to which replicates were assigned to the drought treatment vs. the control treatment because we needed that information to water them accordingly. During the Test Phase we also were not blinded to host species because they are visually distinct. However, we were

blinded to the microbial inoculum treatments during data collection (that information was linked to the replicates' unique ID numbers).

Did the study involve field work? ☒ Yes ☐ No

Field work, collection and transport

Field conditions	We did not record daily weather conditions during our sampling trips.
Location	GPS coordinates for all collection sites are provided in Supplemental Table S1.
Access & import/export	The Kansas soils were collected from pre-existing research sites that were established as part of the U.S. National Science Foundation grant OIA-1656006. Permission was acquired from the corresponding land managers (The Nature Conservancy in Kansas (Smoky Valley Ranch) and the Konza Prairie Biological Station).
Disturbance	A minor amount of soil was removed from each collection site. To minimize disturbance, we collected and pooled several small samples rather than digging a single large hole in any site.

Reporting for specific materials, systems and methods

We require information from authors about some types of materials, experimental systems and methods used in many studies. Here, indicate whether each material, system or method listed is relevant to your study. If you are not sure if a list item applies to your research, read the appropriate section before selecting a response.

Materials & experimental systems

n/a	Involved in the study	
☒	☐	Antibodies
☒	☐	Eukaryotic cell lines
☒	☐	Palaeontology and archaeology
☒	☐	Animals and other organisms
☒	☐	Clinical data
☒	☐	Dual use research of concern
☐	☒	Plants

Methods

n/a	Involved in the study	
☒	☐	ChIP-seq
☒	☐	Flow cytometry
☒	☐	MRI-based neuroimaging

Plants

Seed stocks	Maize genotype B73 was acquired courtesy of Dr. Peter Balint-Kurti (USDA-ARS). Tripsacum dactyloides cultivar "Pete" was purchased from the Gamagrass Seed Company (Falls City, NE, USA).
Novel plant genotypes	N/A
Authentication	N/A
